# Structural constraints of pyocin S2 import through the ferripyoverdine receptor FpvAI

**DOI:** 10.1093/pnasnexus/pgae124

**Published:** 2024-03-27

**Authors:** Jonathan D Goult, Daniel C L Van, Yasmin V Taylor, Patrick G Inns, Renata Kaminska, Martin Vesely, Colin Kleanthous, Emanuele Paci

**Affiliations:** Department of Biochemistry, University of Oxford, Oxford OX1 3QU, UK; Astbury Centre for Structural Molecular Biology, University of Leeds, Leeds LS2 9JT, UK; Astbury Centre for Structural Molecular Biology, University of Leeds, Leeds LS2 9JT, UK; Department of Biochemistry, University of Oxford, Oxford OX1 3QU, UK; Department of Biochemistry, University of Oxford, Oxford OX1 3QU, UK; Department of Biochemistry, University of Oxford, Oxford OX1 3QU, UK; Department of Biochemistry, University of Oxford, Oxford OX1 3QU, UK; Dipartimento di Fisica e Astronomia, Università di Bologna, Bologna 40127, Italy

## Abstract

TonB-dependent transporters (TBDTs) mediate energized transport of essential nutrients into gram-negative bacteria. TBDTs are increasingly being exploited for the delivery of antibiotics to drug-resistant bacteria. While much is known about ground state complexes of TBDTs, few details have emerged about the transport process itself. In this study, we exploit bacteriocin parasitization of a TBDT to probe the mechanics of transport. Previous work has shown that the N-terminal domain of *Pseudomonas aeruginosa*–specific bacteriocin pyocin S2 (PyoS2_NTD_) is imported through the pyoverdine receptor FpvAI. PyoS2_NTD_ transport follows the opening of a proton-motive force-dependent pore through FpvAI and the delivery of its own TonB box that engages TonB. We use molecular models and simulations to formulate a complete translocation pathway for PyoS2_NTD_ that we validate using protein engineering and cytotoxicity measurements. We show that following partial removal of the FpvAI plug domain which occludes the channel, the pyocin's N-terminus enters the channel by electrostatic steering and ratchets to the periplasm. Application of force, mimicking that exerted by TonB, leads to unraveling of PyoS2_NTD_ as it squeezes through the channel. Remarkably, while some parts of PyoS2_NTD_ must unfold, complete unfolding is not required for transport, a result we confirmed by disulfide bond engineering. Moreover, the section of the FpvAI plug that remains embedded in the channel appears to serve as a buttress against which PyoS2_NTD_ is pushed to destabilize the domain. Our study reveals the limits of structural deformation that accompanies import through a TBDT and the role the TBDT itself plays in accommodating transport.

**Significant Statement**

Exclusion of antibiotics by the gram-negative outer membrane (OM) is a crucial mechanism by which *Pseudomonas aeruginosa* evades conventional therapeutics and causes multidrug-resistant infections. However, this seemingly impenetrable defense is bypassed by the significantly larger protein bacteriocins. The mechanisms by which such large molecules traverse the OM have remained elusive. The crystal structure of the bacteriocin pyocin S2 (PyoS2) bound to its OM translocator FpvAI only provides a “snapshot” of the dynamic process of bacteriocin import. In this study, we utilize a combination of computational and biochemical experiments to delineate key steps during the import of PyoS2. PyoS2 is demonstrated to translocate through a pore formed by the FpvAI translocator; threading through in a partially unfolded state and allowing circumvention of the strict selectivity filter imposed on antibiotics by the OM.

## Introduction

*Pseudomonas aeruginosa* is an opportunistic pathogen and major causative agent of multidrug-resistant infections, displaying innate resistance to a large array of conventional antibiotics (1). This is due, in part, to the gram-negative bacterial outer membrane (OM), a protective barrier that functions to prevent the entry of both hydrophilic and hydrophobic compounds into the cell. The *P. aeruginosa* OM contains a large assortment of OM proteins (OMPs) with diverse functions that serve to modulate nutrient and metabolite uptake (2). In addition to the high-level expression of multidrug efflux pumps, *P. aeruginosa* excludes many classes of antibiotics, such as the aminoglycosides, quinolones, and β-lactams (3). However, *P. aeruginosa* has evolved competitive strategies for the exploitation of OMPs to deliver toxins, known as bacteriocins, across the cellular envelope (4). Bacteriocins are protein antibiotics expressed and secreted by bacteria in response to environmental stress, targeting closely related bacterial species and playing a fundamental role in interbacterial competition within microbial communities (4–6) and the pathogenicity of invasive bacteria (7, 8). Bacteriocins are the current focus of concerted efforts for the development of antibiotics to combat multidrug-resistant infections (9, 10).

*P. aeruginosa* synthesize three distinct families of bacteriocins, designated F-, R-, and S-type pyocins, which differ in both structure and mode of cytotoxic activity (11). S-type (soluble) pyocins are structurally and functionally similar to colicins—*Escherichia coli–*specific bacteriocins (4). Both possess modular structures, consisting of N-terminal and middle domains that parasitize proteins within the cellular envelope to deliver their cytotoxic C-terminal domain into target cells. Cytotoxicity is elicited through depolarization of the cell by insertion of a pore, or enzymatic cleavage of peptidoglycan precursors or nucleic acids (DNA, rRNA, and tRNA) (12). Until recently, the mechanisms underpinning pyocin import across the OM were poorly understood, with only a small number of S-type pyocins being functionally characterized. However, developments in structural, microscopic, and in vivo crosslinking studies have significantly advanced our understanding of pyocin import across the OM (13).

The best-studied S-type pyocins are pyocin S2 (PyoS2), pyocin S5 (PyoS5), and pyocin G (PyoG). Like colicins, pyocins exploit a wide variety of cell envelope proteins in order to deliver their cytotoxic domains to their sites of action (14). PyoS2, PyoS5, and PyoG parasitize OMPs from the TonB-dependent transporter (TBDT) family, the ferripyoverdine (FpvAI), ferripyochelin (FptA), and hemin (Hur) receptors, respectively, to translocate across the OM (15–18). TBDTs are required for the energized uptake of specific, often scarce, nutrients from the environment in the form of siderophores, metal chelates, and carbohydrates (19). They are typically composed of two domains: a 22-stranded β-barrel structure that spans the OM and a globular plug domain that occludes the β-barrel domain (20). Unlike colicins, S-type pyocins bind TBDTs through their N-terminal domains (11), with some additionally using the common polysaccharide antigen as their primary OM receptors (21). After binding their specific receptors, an unstructured N-terminal region containing a Ton-binding motif somehow passes through the target transporter. Upon translocation, they expose this binding motif to the periplasm, facilitating contact with a periplasm spanning, proton-motive force (PMF)-linked system within the inner membrane (IM), the Ton complex. The Ton complex is comprised of three IM proteins—TonB/ExbB/ExbD, which are required for energizing nutrient uptake at the OM through coupling to the cellular PMF (20). All components that are parasitized by PyoS2, PyoS5, and PyoG to translocate across the OM are known virulence factors of pathogenic *P. aeruginosa* strains.

The structures of full-length PyoS5 and the N-terminal domain of PyoS2 (PyoS2_NTD_) in complex with its OM translocator FpvAI have been reported (22, 23). Despite displaying minimal sequence homology and binding different TBDT translocators, the N-terminal domains of both pyocins are structurally similar, adopting a kinked three-helix bundle (kTHB) fold, which AlphaFold predictions demonstrate is a common structural motif amongst S-type pyocins (24).

In this study, starting from the FpvAI–PyoS2_NTD_ structure, we generated molecular models and performed dynamic simulations to determine the hypothetical sequence of events for PyoS2 import at atomistic resolution. These models were used to formulate hypotheses and design specific in vitro and in vivo experiments to confirm or refute these hypotheses. We show that upon partial unplugging of the FpvAI transporter, the unstructured N-terminus of PyoS2 positions itself above the newly formed pore. We demonstrate that extensive domain unfolding of PyoS2_NTD_ is required for import through a relatively narrow pore. Nevertheless, disulfide crosslinking experiments show that some local structural features are tolerated as PyoS2 translocates. By demonstrating that complete unfolding is not a prerequisite for PyoS2 transport, a mechanism likely shared with all S-type pyocins, our study also highlights the potential utility of pyocins for the transport of bulky antibiotics into cells, by analogy with siderophore-based transport of cephalosporins through TBDTs (25).

## Results

### Application of force at the N-terminus of FpvAI generates an open channel through partial unfolding the plug domain of the FpvAI–PyoS2_NTD_ complex

PyoS2 translocates through FpvAI, a TBDT that actively transports the siderophore pyoverdine (PVD) in complex with ferric iron (Fe^3+^) into cells (26). FpvAI has typical TBDT architecture: a β-barrel spanning the OM with a central channel occupied by a globular plug domain (27). The plug is remodeled during substrate transport by coupling to the PMF through contacts between FpvAI and the TonB1 complex, facilitating Fe^3+^–PVD uptake (28). FpvAI binds to TonB1 through a TonB1 box located on the periplasmic face of the protein (29). The TonB1 box of FpvAI is known to be required both for Fe^3+^–PVD import and PyoS2 toxicity (21).

**Fig. 1.**
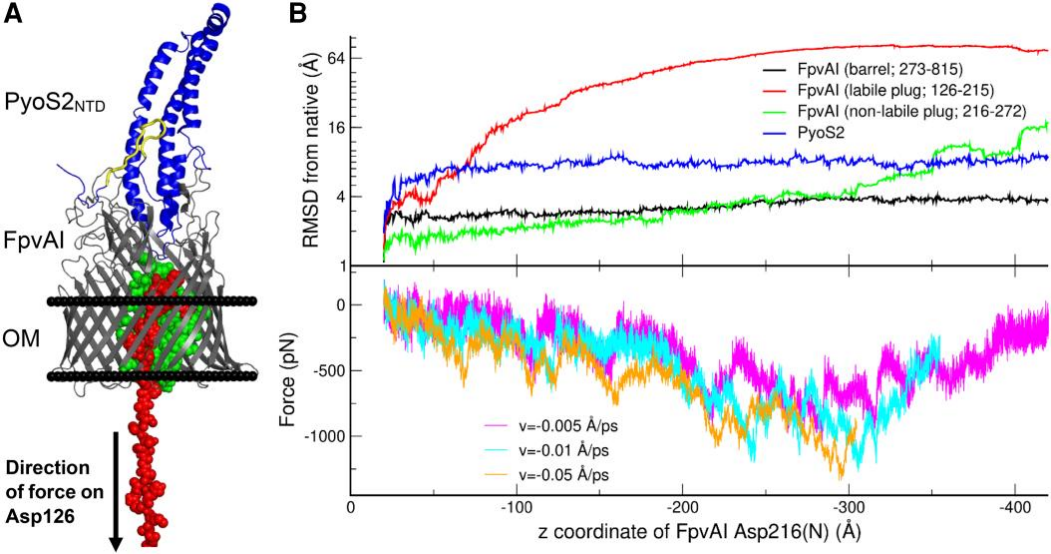
Application of force on the FpvAI N-terminus of the FpvAI–PyoS2_NTD_ complex partially unfolds the plug domain but does not stimulate PyoS2 translocation in silico. A) Snapshot of the structure of PyoS2_NTD_ (blue) in complex with FpvAI (gray) during simulation of force-dependent unfolding of the FpvAI plug domain, highlighting the labile (red) and nonlabile (green) subdomains of the plug domain (from the FpvAI–PyoS2_NTD_ crystal structure [PDB: 5ODW]). The PyoS2 TonB1 box is located within a β-hairpin (yellow) that associates with the N-terminal kTHB. B) Retraction of Asp126 in the −*z* direction (perpendicularly to the membrane toward the periplasm) led to unfolding of the labile plug subdomain (red). The nonlabile plug subdomain (green) starts unfolding when the labile plug is completely extended (∼300 Å for the 90 residues that constitute the labile plug). Unfolding of the labile plug subdomain starts at low forces, while the initiation of the unfolding of the nonlabile plug subdomain corresponds to a peak in the force (shown for three different trajectories at different pulling speeds: magenta, cyan, and yellow). Removal of plug domain does not affect the structure of the FpvAI β-barrel (black), or cause remodeling of PyoS2_NTD_ (blue), suggesting that unplugging of the transporter and translocation of pyocin do not occur concomitantly.

The FpvAI–PyoS2_NTD_ crystal structure captures FpvAI in a closed state (22), with the β-barrel completely occluded by the plug domain (Fig. 1A). To mimic the TonB1-mediated forced extraction of the plug domain of the FpvAI–PyoS2_NTD_ complex, we performed (steered) molecular dynamic simulations. The periplasmic signaling domain (residues 1 to 125, which includes the secretion signal) of FpvAI was removed, and an ideal spring was attached to residue Asp126. The spring was then moved at constant velocity perpendicularly to the membrane in the direction of the periplasm. The deformation of the spring is proportional to the force with which the polypeptide chain responds to the retraction of the spring. This setup mimics the force exerted by TonB1 on the FpvAI TonB1 box in the periplasm (Fig. 1B). Simulations showed that the FpvAI plug domain unfolds as two structurally independent entities: the N-terminal portion of the chain up to Asn215 unfolds at relatively low force and unravels entirely, before a second portion of the plug (residues 216 to 272) unfolds at considerably larger force. The second portion of the plug starts unfolding once the former is fully extended (when the N-terminal residue of FpvAI is at a distance of ∼300 Å from the center of the membrane) and a peak in the force is observed, characteristic of “mechanically stable” proteins (30, 31) (Fig. 1B). The existence of a TBDT plug with two mechanically distinct subdomains is supported by previous simulations and force spectroscopy measurements (32, 33). The labile subdomain is proximal to the TonB1 box that in vivo would be the point of force delivery by TonB1. The FpvAI β-barrel domain and the nonlabile subdomain of the plug preserve their structural integrity throughout the simulation (Fig. 1B). During the extraction of the labile subdomain, PyoS2_NTD_ remained folded and bound to FpvAI (Fig. 1B). This suggests that the PyoS2_NTD_ is “mechanically independent” from FpvAI, at least on the timescales explored by these simulations, which are considerably shorter than those in which TonB1-mediated unplugging of FpvAI likely occurs in vivo. Extraction of the labile subdomain of FpvAI reveals a pore spanning the OM, with the nonlabile plug subdomain remaining associated within the β-barrel and forming a portion of the pore wall (Fig. 1B).

To explore the role of the FpvAI plug in PyoS2 translocation in vivo, we utilized a *P. aeruginosa* strain, PAS033, expressing the FpvAI β-barrel domain only. Strains expressing the FpvAI β-barrel domain alone have previously been shown to be deficient in both Fe^3+^–PVD binding and uptake (29). However, the interface between PyoS2 and the FpvAI β-barrel domain is significantly more extensive than that of the native ligand (22, 27) and thus PyoS2 could potentially parasitize a plugless transporter. A chimeric PyoS2E2 construct (used to subvert PyoS2 resistance within the *P. aeruginosa* parent strain) was found to be inactive against PAS033 (Fig. S1A). Therefore, PyoS2 is unable to translocate through the plugless FpvAI transporter. This was further investigated through fluorescence microscopy using a fluorescently labeled PyoS2_NTD_ (PyoS2_NTD_-AF488) construct to probe interactions with the plugless FpvAI transporter. PAS033 resistance to chimeric PyoS2E2 activity was demonstrated to stem from the abrogation of binding between the plugless FpvAI transporter and the N-terminal domain of PyoS2. PyoS2_NTD_-AF488 was unable to specifically label PAS033 compared with the PAO1 wild-type strain (Fig. S1B and C). The FpvAI plug domain constitutes a sizeable interaction surface for the PyoS2 N-terminus, comprising ∼10% of the overall interface surface area between the two proteins in the bound conformation. Loss of these interactions via deletion of the FpvAI plug abolishes this interaction surface between the transporter and the PyoS2 N-terminus, which likely explains the lack of PyoS2 binding.

The expression of a soluble FpvAI plug domain (*fpvAI_1-276_*) has previously been shown to complement the plugless FpvAI β-barrel and partially restore Fe^3+^–PVD uptake in PAS033 cells (29). Transformation of PAS033 with a plasmid encoding the soluble FpvAI plug domain restored susceptibility to PyoS2E2, indicating that pyocin translocation can also be re-established *in-trans* (Fig. S1A and D). Quantification of fluorescent labeling of PAS033 cells expressing the soluble FpvAI plug domain confirmed restoration of PyoS2_NTD_ binding, albeit at a reduced level compared with PAO1 cells expressing full-length FpvAI (Fig. S1B and C). This reduction in fluorescence intensity, which constitutes both bound and translocated PyoS2_NTD_-AF488, is analogous to the reduced rate of Fe^3+^–PVD uptake for the reconstituted transporter shown previously (29). The observed reduction in PyoS2 (and Fe^3+^–PVD) transporter efficacy for the reconstituted FpvAI transporter likely occurs due to competition for TonB1 binding between the periplasmic pool of soluble FpvAI plug and those associated with the reconstituted transporter in vivo (Fig. S1C and D). As our simulations demonstrate that the FpvAI nonlabile subdomain of the plug remains associated with the inner wall of the β-barrel during unplugging, we evaluated whether this subdomain alone could restore binding and translocation of PyoS2. A truncation of the soluble plug domain, constituting the nonlabile subdomain only (*fpvAI_216-276_*), was generated and transformed into PAS033. However, cells expressing the nonlabile plug subdomain remained resistant to both PyoS2E2 activity and PyoS2_NTD_-AF488 labeling (Fig. S1A–C). We conclude that under these experimental conditions, an intact FpvAI plug domain is a requirement for PyoS2 translocation across the OM in vivo, even though a portion of the plug is removed during PyoS2 transport.

### Electrostatic interactions likely direct the PyoS2 TonB1 box through an open FpvAI pore

**Fig. 2.**
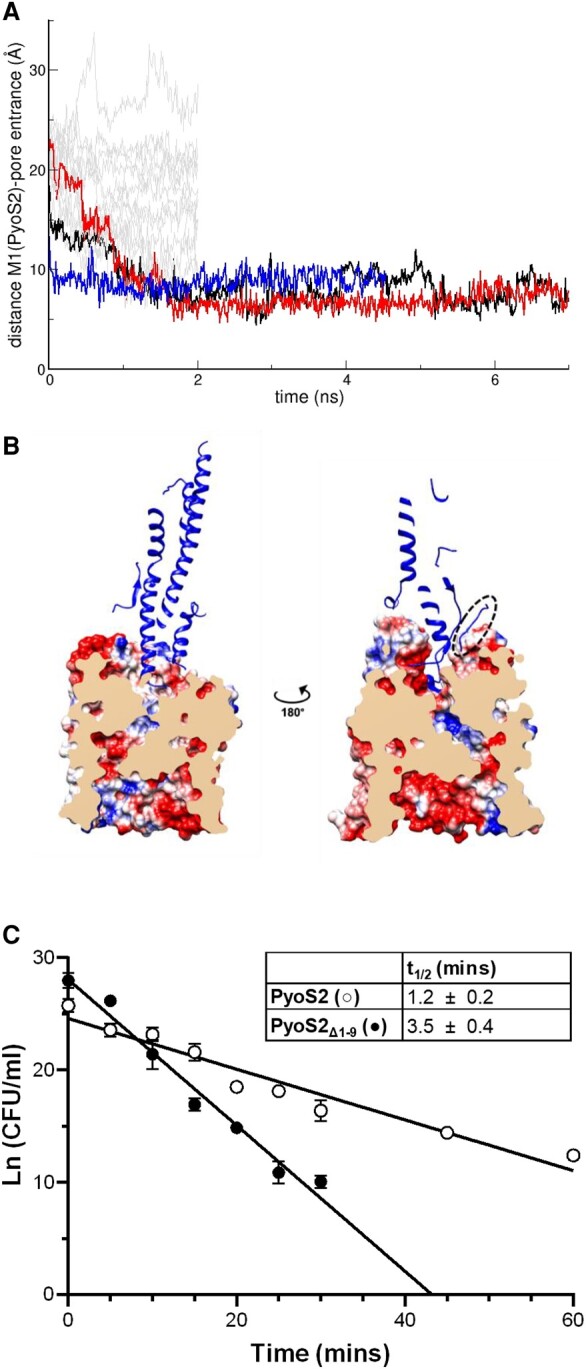
The unstructured N-terminus of PyoS2 is required for optimal import. A) Distance between the (modeled) main chain N of Met1 of PyoS2 from the FpvAI pore entrance demonstrates the disordered N-terminal end of PyoS2 can spontaneously diffuse to the entry of the pore vacated by the unfolded labile plug. Running simulations from random conformations of the N-terminal unstructured residues of PyoS2 reveal a small number of cases in which the N-terminus is observed diffusing toward the pore (identified as the center of mass of residues Val229, Trp707, and Thr797). B) Transverse of FpvAI receptor (surface) with PyoS2_NTD_ bound (blue). Removal of the labile plug domain leaves a pore spanning the membrane through the β-barrel. The pore lining is composed of alternating charges and the unstructured PyoS2 N-terminus (dashed oval) is positioned near the pore entrance, as described previously. The unfolded labile plug domain was removed from this analysis and subsequent simulations. C) PyoS2 kinetic time-course cytotoxicity assay demonstrates that truncation of unstructured residues upstream of the TonB1 box decreases the rate of translocation for PyoS2Δ1-9 (○) compared with PyoS2 (●). Colony counts from four independent YHP17 cultures (with error bars representing SD) were fitted to a pseudo-first order reaction model, and translocation half-lives (*t*_1/2_) were calculated from the linear plot.

S-type pyocins engage the PMF through their N-terminal TonB1 box, which drives transport across the OM (22, 23). In the FpvAI–PyoS2_NTD_ complex, the PyoS2 TonB1 box is sequestered in a β-hairpin that is associated with the kTHB domain (Fig. 1A). To energize uptake, the TonB1 box of PyoS2 must dissociate from this position and enter the periplasm, where it can contact TonB1. However, the β-hairpin motif is ∼33 Å from the entrance to the FpvAI pore in our simulations (Fig. 1A). The residues preceding the PyoS2 TonB1 box are presumed disordered, as they are not resolved in the FpvAI–PyoS2_NTD_ crystal structure. The importance of disordered protein regions in bacteriocin translocation across the OM has previously been highlighted (34, 35). We hypothesized that the unstructured N-terminus of PyoS2 might play a role in the delivery of the PyoS2 TonB1 box to the periplasm. To explore this possibility, the unstructured N-terminus of PyoS2 was modeled using MODELLER (36). Molecular dynamic simulations were carried out starting from the 50 lowest energy models. In a subset of these simulations, the N-terminal residue of PyoS2 spontaneously positioned itself above the pore entrance of FpvAI, “priming” it for translocation (Fig. 2A and B). This spontaneous positioning above the open pore is likely driven by electrostatic interactions between the FpvAI pore entrance and the unstructured N-terminus of PyoS2; the unstructured region of PyoS2 contains several negatively charged amino acids (pI = 3.67), while the entrance to the FpvAI pore is positively charged (pI = 8.76).

Alignment of the PyoS2 unstructured N-terminus with other known TonB1-dependent pyocins suggests similarly charged regions preceding the TonB1 box are observed in other S-type pyocins. All sequences analyzed possess an acidic/hydrophobic amino acid pair preceding their putative TonB1 boxes, though the precise distance from the pentapeptide sequence varies and are overall electronegatively charged in the majority of S-type pyocin N-terminal sequences (Fig. S2A). Inspection of the pore walls formed by the FpvAI β-barrel and nonlabile plug subdomain during our simulations revealed distinct alternating patches of charge on one face, in addition to a hydrophobic face (Fig. 2B). We hypothesized that these complimentary charges may be involved in facilitating directed diffusion of the N-terminus, and its associated TonB1 box, through the pore and into the periplasm.

To evaluate whether the unstructured N-terminus plays a role in PyoS2 translocation in vivo, residues preceding the TonB1 box of PyoS2 were truncated (PyoS2Δ1–9) and the resulting construct tested for cytotoxic activity. PyoS2Δ1–9 displayed no significant reduction of cytotoxicity against *P. aeruginosa* strain YHP17 in overlay assays that involve overnight incubation with the toxin (Fig. S2B). However, kinetic time-course cytotoxicity experiments (following changes in colony-forming units [CFU] over time) showed that the PyoS2Δ1–9 deletion had a 3-fold impact on the half-life for killing; *t*_1/2_ = 1.2 ± 0.2 min for PyoS2 compared with 3.5 ± 0.4 min for PyoS2Δ1–9. Previous work on *E. coli*–specific bacteriocins has shown that the pseudo-first order kinetic profiles typical of these toxins are due to the PMF-dependent, rate-limited transport of the bacteriocin across the OM (37). Hence, the kinetic penalty in PyoS2 killing that results from the loss of the N-terminal residues preceding the TonB1 box is consistent with a role in initiating the import process.

### Substantial remodeling of the PyoS2 N-terminus must occur in order to translocate through the FpvAI pore

**Fig. 3.**
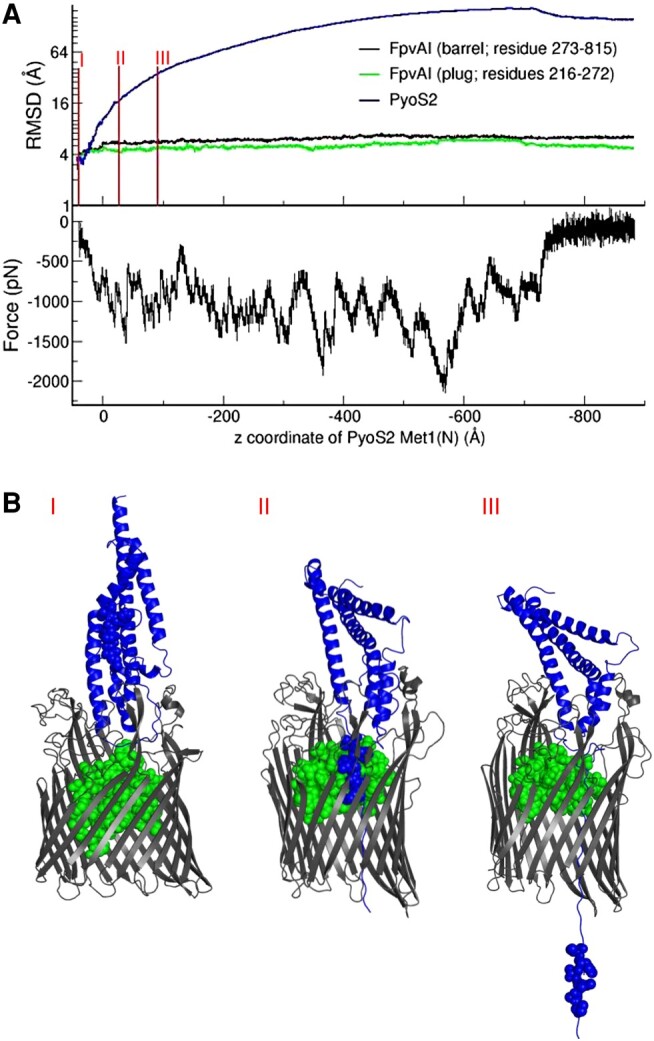
The N-terminal domain of PyoS2 must unfold in order to translocate through the pore formed by the FpvAI transporter. A) The rmsd of the different structural components (top) and force applied (bottom) during translocation of PyoS2_NTD_. PyoS2_NTD_ readily unfolds and displaces from its original position upon retraction of Met1 of PyoS2 in the −*z* direction. FpvAI barrel and nonlabile plug undergo minimal perturbance in response to PyoS2_NTD_ translocation. B) Snapshots of PyoS2 during the forced translocation shown as blue spheres (see Movie S1). Vertical lines in A) (I, II, and III) correspond to the time at which the snapshots were taken.

For bacteriocins that exploit porins in *E. coli*, OM translocation requires complete unfolding in order to thread through the narrow pores of the porin (37, 38). It has previously been shown that the addition of disulfide bonds to bacteriocins arrests translocation and inhibits cytotoxicity (39, 40). The pore through which porin-dependent bacteriocins translocate is too narrow to accommodate an intramolecular crosslink. In vivo crosslinking experiments have demonstrated that PyoS2 crosslinks to residues within the FpvAI barrel, suggesting that it must pass through the pore created by the removal of the labile plug subdomain (22). However, in what form the folded pyocin is transported remains unknown. To establish an initial starting point for mechanistic experimental investigations, we simulated the translocation of PyoS2 by applying an external force perpendicularly to the membrane on the N-terminal residue of PyoS2_NTD_ positioned above the FpvAI pore. MD simulations demonstrated that the PyoS2 N-terminal domain readily unfolds as it is pulled through the pore formed by FpvAI (Fig. 3A and B, Movie S1). Translocation does not perturb the FpvAI β-barrel domain and has minimal impact on the FpvAI nonlabile plug subdomain, which remains associated with the barrel during translocation. The rationale for applying a force in the direction of the periplasm to the PyoS2 N-terminus is different from that used to unplug the transporter FpvAI. In the latter simulations, the applied force mimics the application of force provided by TonB1, whereas force application on the N-terminus of PyoS2 ultimately covers two separate translocation events—the spontaneous diffusion of the unstructured N-terminus through the FpvAI pore, and the TonB1-dependent energized translocation of the remaining PyoS2 N-terminal domain. While our simulations amalgamate these two events, they confirm that minimal structural disruption occurs to the FpvAI transporter upon force-directed translocation of the PyoS2_NTD_ through the open pore. The pore resulting from the extraction of the labile domain is sufficiently wide for PyoS2_NTD_ to translocate across FpvAI but seemingly not wide enough for the preservation of any local structure. Hence, it is expected that disulfide bonds would prevent translocation through the pore, unless the FpvAI transporter loses its structural integrity.

**Fig. 4.**
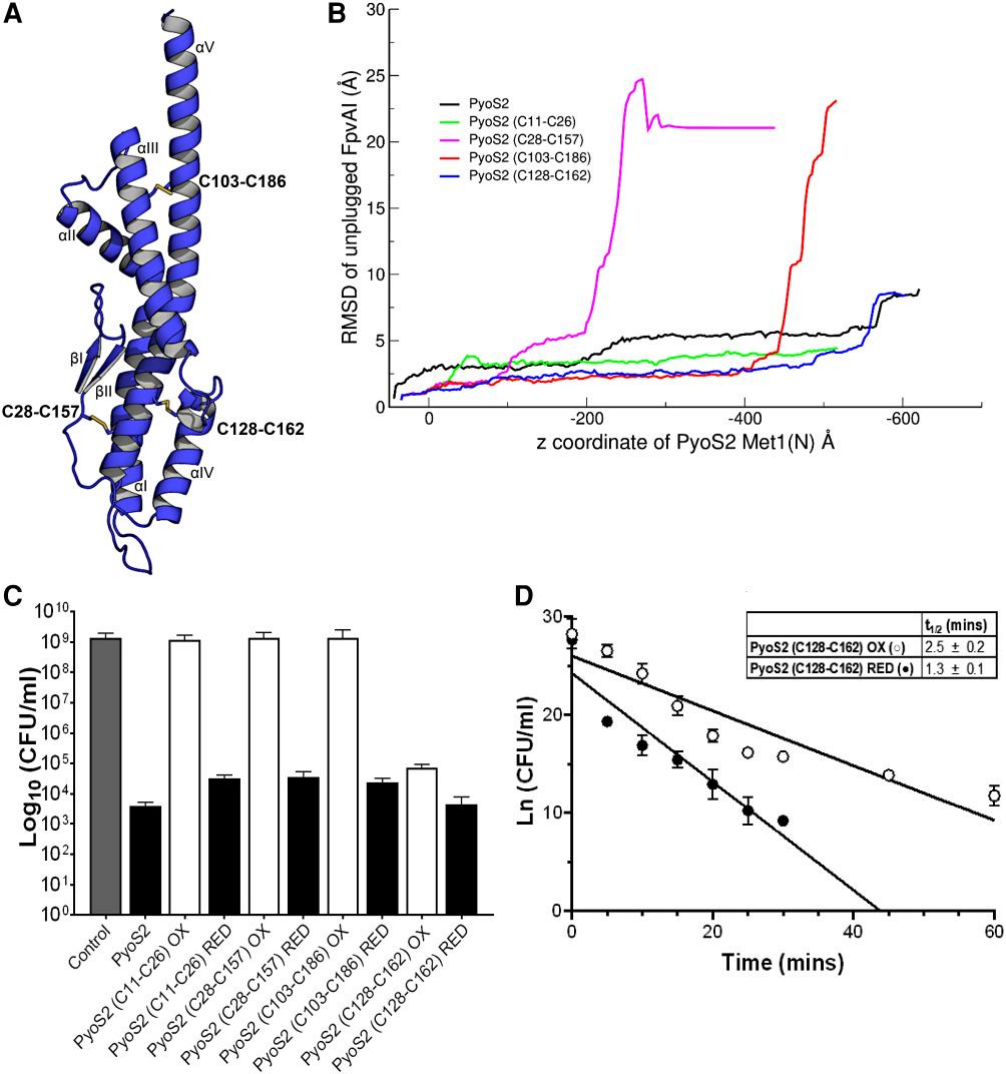
Disulfide bonds impede, but not necessarily prevent, translocation of PyoS2 through the pore formed by the FpvAI transporter. A) Depiction of PyoS2_NTD_ with disulfide positions predicted by Disulfide by Design (disulfide C11–C26 not shown). B) The rmsd of the unplugged transporter FpvAI as a function of the *z*-coordinate of the first residue of PyoS2. Two variants (C28–C157 and C103–C186) result in a severe structural change in the transporter, while in the case of C11–C26 and C128–C162, the disulfide bond can go through the pore causing only minor structural rearrangements (see Movies S2–S5). C) YHP17 CFU/mL from PyoS2 cytotoxicity assay after 3 h shows disulfide positions C11–C26, C28–C157, and C103–C186 inhibit PyoS2 cytotoxicity when oxidized (white). This effect is reversed upon disulfide bond reduction (black). Disulfide position C128–C162 imparts significantly reduced protection against PyoS2 activity when oxidized. Colony counts from six independent cultures with error bars representing the SD. D) YHP17 CFU/mL from kinetic time-course cytotoxicity assays demonstrate that oxidation of the C128–C162 disulfide bond results in a decrease in the PyoS2 translocation rate (○) compared with that of the reduced form (●). Colony counts from four independent cultures (with error bars representing SD) were fitted to a pseudo-first order reaction model and translocation half-lives (*t*_1/2_) were calculated from the linear plot.

To probe the structural deformations of PyoS2_NTD_ during transport, we introduced cysteine mutations into the PyoS2 N-terminal domain at various positions that were predicted to form disulfide bonds by Disulfide by Design (https://doi.org/10.1186/1471-2105-14-346) (41) (Fig. 4A). MD simulations of the forced translocation of the oxidized disulfide mutants of PyoS2_NTD_ show broadly diverse trajectories, and in all cases, the structure of the transporter is disrupted (nonlabile plug subdomain and/or the β-barrel). However, this damage is unlikely to happen at the pulling speeds and force ranges occurring in vivo. We then performed simulations at zero temperature—which provides an approximate minimum energy pathway that depends weakly on the pulling speed. In two cases (disulfides C28–C157 and C103–C186), the whole nonlabile plug subdomain is displaced from the barrel once the disulfide bond engages with the FpvAI pore (Fig. 4B, Movies S3 and S4). However, for disulfides C11–C26 and C128–C162, the FpvAI transporter undergoes moderate perturbations during the translocation (Fig. 4B, Movies S2 and S5). Hence, the simulations show that the FpvAI transporter pore could potentially accommodate a disulfide crosslink if the intervening region between the two cysteines is unstructured, which is not the case for the two variants (C28–C157 and C103–C186) in which the pair of cysteines are furthest apart in sequence.

We evaluated the effects of these disulfides on PyoS2 transport by generating the relevant disulfide mutants in PyoS2. Disulfide bond formation was confirmed using an in vitro fluorescence-based assay in which oxidized and reduced PyoS2 disulfide mutants were labeled with AlexaFluor^488^ under denaturing conditions (Fig. S3). Additionally, the N-terminal domains of the PyoS2 disulfide mutants were also expressed in isolation to assess whether disulfide formation affected domain structure and stability. For the N-terminal disulfide mutants that could be expressed, all possessed identical secondary structure to PyoS2_NTD_ and the variation in thermal stability of the domain was moderate (*T*_m_ = 44–50 °C) compared with PyoS2_NTD_ (Fig. S4).

As delivery of the PyoS2 TonB1 box to the periplasm is required to stimulate energized translocation, disulfide bonds were engineered to lock the β-hairpin motif of PyoS2, (PyoS2(C11–C26) and PyoS2(C28–C157)), preventing β-strand separation, and hairpin displacement from the kTHB domain, respectively. Oxidized PyoS2(C11–C26) and PyoS2(C28–C157) mutants were inactive against *P. aeruginosa* YHP17 (Figs. 4C and S5A, B). Reduction and alkylation of free cysteines to prevent disulfide bond reformation restored PyoS2 cytotoxic activity for both mutants (Fig. 4C and S5A, B). This confirms that TonB1 box delivery to the periplasm requires flexibility within the β-hairpin; the β-hairpin must displace from the α-helical bundle and the β-strands must separate and pass through the FpvAI pore in an extended conformation. This observation contradicts translocation simulations of the C11–C26 disulfide, which translocates through the FpvAI pore in silico. However, as highlighted above, these simulations are force directed and as such, cannot evaluate the initial spontaneous diffusion of the N-terminal leader sequence through the FpvAI pore, prior to contacting TonB1. Therefore, without application of an external force to drive import of the disulfide, oxidized PyoS2(C11–C26) is unable to translocate and elicit cytotoxicity in vivo.

To establish the degree of PyoS2_NTD_ unfolding during import, disulfide bonds were engineered at two different positions within the kTHB domain (PyoS2(C103–C186) and PyoS2(C128–C162)). Oxidized PyoS2(C103–C186) mutant inhibited pyocin cytotoxicity (Figs. 4C and S5C) against *P. aeruginosa* YPH17. Reduction (and alkylation) of PyoS2(C103–C186) fully restored cytotoxic activity (Figs. 4C and S5C), suggesting that the disulfide blocks translocation by impeding domain unfolding. However, oxidized PyoS2(C128–C162) remained active against *P. aeruginosa* YP17 (Figs. 4C and S5D), as suggested by the MD simulations. We further investigated this mutant utilizing kinetic time-course cytotoxicity experiments to identify whether the disulfide impacted PyoS2 translocation kinetics through the FpvAI pore. Oxidized PyoS2(C128–C162) displayed slower cell-killing kinetics relative to the reduced mutant (Fig. 4D); half-lives (*t*_1/2_) of translocation for oxidized PyoS2(C128–C162) of 2.5 (±0.2) min compared with reduced PyoS2(C128–C162) of 1.3 (±0.1) min. Hence, although an internal crosslink within PyoS2_NTD_ can be accommodated, it nevertheless impacts transport kinetics through the partially unplugged FpvAI transporter.

## Discussions

**Fig. 5.**
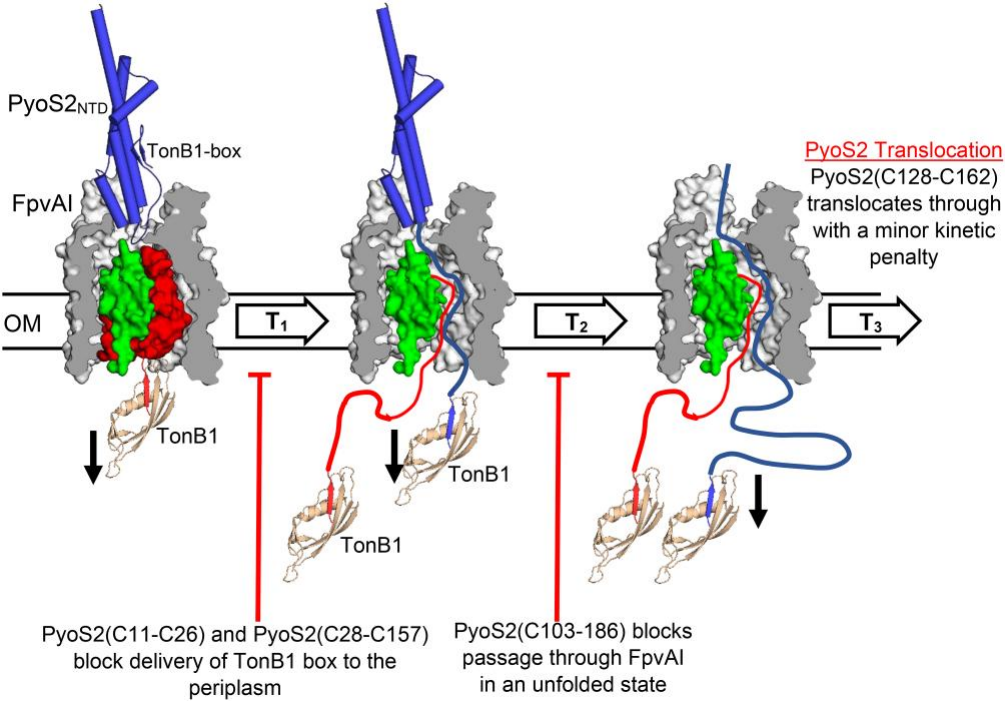
Updated model of PyoS2 import through FpvAI and the structural constraints imposed during translocation. A three-step model for PyoS2_NTD_ import through the FpvAI transporter, supported by in silico, in vitro, and in vivo experimental data presented here. Initial binding of PyoS2_NTD_ to FpvAI mimics Fe^3+^–PVD binding, activating the transporter for uptake and recruiting TonB1 via its TonB1 box. Step 1 (T_1_): Force remodeling of the labile portion (red) of the FpvAI plug domain occurs in a PMF-dependent manner via contact to the TonB1–ExbB–ExbD complex in the IM (only the C-terminus of TonB1 is shown for clarity). The N-terminus of PyoS2_NTD_ enters the resultant pore formed, facilitating presentation of its own TonB1 box in the periplasm. This process is blocked by disulfides within the β-hairpin motif. Step 2 (T_2_): The PyoS2_NTD_ TonB1 box is bound by another copy of TonB1, resulting in the force remodeling of PyoS2_NTD_ itself and driving translocation through the FpvAI lumen. Translocation can be stalled by the presence of intramolecular crosslinks in the form of disulfide bonds (C103–C186). Step 3 (T_3_): The entire PyoS2_NTD_ is translocated through the FpvAI lumen, presumably followed by the remainder of the protein for the full-length construct. The PyoS2(C128–C162) mutant can successfully translocate across the OM despite the presence of an intramolecular crosslink, however, import as slowed as a result.

Through a combination of in silico, in vitro, and in vivo experiments, we have delineated a new model for PyoS2 translocation through its TBDT FpvAI and the structural constraints imposed on this process (Fig. 5). Our MD simulations show that TonB1-dependent force remodeling of the FpvAI plug domain results in partial unplugging of the transporter, supporting a model of TBDT unplugging that has previously been demonstrated for *E. coli* BtuB (33). Removal of the labile plug domain does not trigger PyoS2 import, however, indicating unplugging and PyoS2 translocation are mechanically independent of one another. An intact FpvAI plug domain was also shown to be required for PyoS2 translocation in vivo, with reconstituted FpvAI β-barrel and plug domains restoring PyoS2 import in trans. Previous studies on the precise role that TBDT plug domains play in bacteriocin transport are conflicting, with TBDT plug deletions inhibiting some bacteriocins (42, 43), yet others appear to be unaffected (44). From our experiments, it is apparent that the presence of the intact FpvAI plug domain is a prerequisite for PyoS2 translocation in vivo (45). However, the precise role that the FpvAI nonlabile portion of the plug plays on the import process has been unclear. Our MD simulations suggest that the nonlabile subdomain undergoes relatively small and reversible perturbations during low energy pathway simulations (at a range of pulling speeds) of PyoS2_NTD_ translocation through the FpvAI pore. While we could not measure a correlation between the advancement of PyoS2_NTD_ through the pore and the reversible movements of the nonlabile plug, it is possible that this movement functions as a “ratchet” that prevents backward diffusion, which has been proposed previously (4).

Simulations suggested, and experiments confirmed, that the unstructured N-terminus of PyoS2 “primes” the toxin for import by positioning itself above the open FpvAI pore. Unstructured N-terminal regions have previously been shown to be important for the presentation of binding motifs in the periplasm prior to energized bacteriocin import (38, 46). The PyoS2 unstructured N-terminus is instead required for orientation of the N-terminal residue at the entrance to the pore, likely assisting in directing diffusion into the pore. This is in agreement with MD (Rosetta) simulations of the translocation of colicin B through its transporter FepA, in which the unstructured N-terminus of the bacteriocin occupies the pore generated upon removal of the FepA labile plug subdomain (47).

The mechanism by which the N-terminal region of PyoS2 diffuses through the pore, or how diffusion in the direction of the periplasm is favored, remains unclear. The conservation of electronegative charge across the majority of unstructured N-termini of the TonB1-dependent pyocins investigated in this study implies that electrostatics might be required for driving diffusion of some, if not all, S-type pyocin N-termini through their transporters prior to energized uptake via contact with the TonB1 complex. The distribution of charged residues lining the FpvAI pore could function to electrostatically “steer” the PyoS2 N-terminus to the periplasm.

Simulations of PyoS2_NTD_ import suggest that the N-terminal domain must unfold during translocation through FpvAI. This process does not significantly perturb the structure of the pore formed by the β-barrel domain and nonlabile plug subdomain, with PyoS2 passing through in an extended conformation. However, attempts to arrest translocation within our simulations through the introduction of disulfides within the PyoS2 N-terminal domain demonstrated greater tolerance for local structural conservation during import than expected. Two of the disulfide positions tested (C11–C26 and C128–C162) were able to translocate through the FpvAI pore, generating marginal perturbations within the nonlabile plug subdomain. Upon further investigation of these disulfide positions in vivo, PyoS2(C128–C162) remained active against *P. aeruginosa*, only suffering a minor kinetic penalty during import, despite the presence of an intramolecular crosslink. The ability of a partially folded substrate to pass through the FpvAI pore infers a degree of tolerance for the preservation of local structural elements during translocation. However, this can only occur after import is energized through contact with the TonB1 complex. Oxidized PyoS2(C11–C26) remains inactive against *P. aeruginosa* despite simulations suggesting translocation was viable through the FpvAI pore in a partially folded state. This is likely due to the inability of the disulfide-locked β-hairpin to spontaneously diffuse through FpvAI in the absence of an applied force, as in our simulations.

Both the arrest of bacteriocin translocation in response to disulfide formation (39, 48) and translocation of bacteriocins containing intramolecular disulfide crosslinks (48) have been demonstrated previously, suggesting significant variation in the structural plasticity of bacteriocin import through their requisite transporters. Previously, Lukacik et al. (48) demonstrated that despite addition of disulfides bonds within the C-terminal cytotoxic domain of an engineered bacteriocin, the toxin remained active against cells expressing its target TBDT receptor, implying translocation can occur without complete unfolding. For the mechanism of PyoS2 translocation investigated in this study, it is evident that only partial unfolding of the PyoS2_NTD_ during import is required, with maintenance of local structural elements tolerated for some regions within PyoS2 (C128–C162), whereas others must translocate in a fully extended conformation (C103–C186).

## Materials and methods

### Molecular modeling

Simulations were performed with a united atom (CHARMM19 (49)), and implicit solvent/membrane model EEF1/IMM1 (50), using the program CHARMM (51); the choice for such a model was based on the size of the system, and the large conformational changes explored in this work. The crystal structure at 2.8 Å resolution of PyoS2_NTD_ bound to FpvAI (Protein Data Bank [PDB]: 5ODW) was used as the initial conformation (22). PyoS2_NTD_ was missing density from residues 1–10, suggesting that the N-terminus is disordered. Chains A and C in 5ODW corresponding to one biological unit of the FpvAI–PyoS2_NTD_ complex were oriented relatively to the membrane using OPM (52); also estimated by OPM, the thickness of the membrane was 23.8 Å. The membrane was positioned perpendicular to the *z*-axis and symmetrically around *z* = 0. Residues 45–125 of FpvAI on the periplasmic side were removed to limit the size of the system. Residues 1–10 of PyoS2 were modeled using MODELLER (36).

Removal of the plug of FpvAI and translocation of PyoS2 were accelerated by linking the main chain carbon to an ideal spring with elastic constant 100 pN/Å and retracting this at constant velocity in the periplasm direction (−*z*), in a range of pulling speeds. The range is limited by computational power, and orders of magnitude faster that the natural TonB1-mediated process. Pathways depend on the pulling speed (or, equivalently, magnitude of force applied); we performed simulations in a range of pulling speeds between 1 and 0.005 A/ps; at the lower pulling speeds, the sequence of events observed converges as evident from force-extension profiles showing similar patterns (e.g. Fig. 2A).

### Secondary structure prediction and sequence alignments

Secondary structure prediction of TonB1-dependent pyocins utilized PSIPRED (53). The unstructured N-termini was identified and defined as the residues preceding the first predicted α-helix. Alignment of the unstructured N-termini was performed using Clustal Omega and conserved residues were identified using BoxShade. Theoretical isoelectric point's (pI) were calculated from sequence alone as described in Bjellqvist et al. (54).

### Bacterial strains

*E. coli* and *P. aeruginosa* strains used in this study are listed in Table 1. Liquid cultures of bacteria were routinely grown by incubation in Lysogeny Broth (LB) media at 37°C unless stated otherwise. Single-colony isolates were generated (when required) by plating cultures onto 1.5% (w/v) LB-agar plates and incubated overnight at 37°C. The *P. aeruginosa* strains used for screening cellular expression of FpvAI derivatives were grown overnight at 30°C in an iron-deficient succinate medium (6 g/L K_2_HPO_4_, 3 g/L KH_2_PO_4_, 1 g/L (NH_4_)_2_SO_4_, 0.2 g/L MgSO_4_·7H_2_O, 4 g/L succinic acid). Antibiotic supplementation with ampicillin (100 μg/mL), carbenicillin (150 μg/mL), and chloramphenicol (10 μg/mL) was used when appropriate to ensure specific bacterial growth.

**Table 1.** Bacterial strains used in this study.

Bacterial strain	Relevant characteristics	Source or reference
*P. aeruginosa strains*		
PAO1	Parent strain	Washington Library
YHP17	Clinical isolate	Walker Lab
PAS033	*pvdF*::kana *fpvA_Δ139-276_*	(29)
*E. coli strains*		
NEB5α	huA2 D(argF-lacZ)U169 phoA glnV44 f80D(lacZ)M15 gyrA96 recA1 relA1 endA1 thi-1 hsdR17	New England Biolabs
BL21 (DE3)	fhuA2[lon], ompT, gal(λDE3)[dcm], Δhsd5	New England Biolabs
S17-1λpir	pro thi hsdR recA; chromosomal RP4 (Tra + Tcs Kms Aps); Tpr Smr	American Type Culture Collection (55)

### Plasmids and site-specific mutagenesis

Plasmid constructs containing *pyoS2* and *fpvAI_1-276_* were provided by the Kleanthous and Schalk Labs, respectively. Novel plasmids containing site-specific mutant inserts are listed in Table 2 and were generated using standard cloning protocols for PCR, restriction digestion, ligation, and transformations.

**Table 2.** Plasmids used in this study.

Plasmid name	Source	Parent vector	Insert
pPW06	(22)	pET21(a+)	*pyoS2-imS2*
pPW11	(22)	pET21(a+)	*pyoS2_NTD_*
pJDG23	This study	pET21(a+)	*pyoS2_NTD_-GSG-Cys*
pJDG07	This study	pET21(a+)	*pyoS2(K103C;L186C)-imS2*
pJDG10	This study	pET21(a+)	*pyoS2(P28C;D157C)-imS2*
pREN120	This study	pET21(a+)	*pyoS2(M11C;Y26C)-imS2*
pREN121	This study	pET21(a+)	*pyoS2(Y128C;E162C)-imS2*
pJDG15	This study	pET21(a+)	*pyoS2Δ1-9-imS2*
pJDG18	This study	pET21(a+)	*pyoS2_NTD_(M11C;Y26C)*
pJDG20	This study	pET21(a+)	*pyoS2_NTD_(K103C;L186C)*
pJDG21	This study	pET21(a+)	*pyoS2_NTD_(Y128C;E162C)*
pMMB-SD-TB-Plug	(29)	pMMB190	*fpvAI_1-276_*
pMMB-Plug_NL_	This study	pMMB190	*fpvAI_Δ45-215_*

### Transformation of *P. aeruginosa*

*E. coli* strain S17-1λpir transformed with pMMB190-derived plasmids, and *P. aeruginosa* strain PAS033 cells were incubated overnight in LB at 37 and 43°C, respectively, without antibiotic selection. Two milliliters of both bacterial cultures were mixed and pelleted by centrifugation (6,800 × *g*, 6 mins) followed by resuspension in 100 μL LB. Cellular suspension was spotted onto a prewarmed LB-agar plate followed by incubation at 37°C for 8 h. *P. aeruginosa* transformants were then selected for on LB-agar plates containing 150 μg/mL carbenicillin and 10 μg/mL chloramphenicol.

### Immunoblot analysis

*P. aeruginosa* cultures were grown in an iron-deficient succinate medium overnight as described previously. Cells were pelleted by centrifugation (5050 × *g*, 12 min) and resuspended in 10 mM Tris-HCl pH 8.0 buffer containing 1 mM phenylmethylsulfonyl fluoride (PMSF). Cells were lysed by sonication on ice (MISONIX S-4000), followed by centrifugation (12,500 × *g*, 30 min, 4°C) to remove insoluble debris. Cellular lysates were run on a 12% sodium dodecyl sulfate (SDS) polyacrylamide gel followed by blotting onto Sequi-Blot polyvinylidene fluoride membrane. The blot was blocked with 8% Marvel dried skimmed milk in Tris-buffered saline buffer with Tween 20 (TBST buffer) overnight at room temperature. The membrane was washed with TBST buffer (5 × 1 min) and probed with primary rabbit α-FpvAI Antiserum (1:250) provided by the Schalk Lab in 4% Marvel milk in TBST for 1 h at room temperature. Membrane was washed as above, then probed with secondary goat αRabbit IgG conjugated to horseradish peroxidase (1:1,000) for 1 h at room temperature. Membrane was washed as above for a final time, followed by detection using Amersham ECL Western Blotting Select Detection Reagent in a GBOX-CHEMI-XRQ.

### PyoS2 mutant expression and purification

Overnight cultures of BL21 (DE3) cells transformed with PyoS2 mutant plasmids were inoculated into sterile LB (1:100) containing 100 μg/mL ampicillin and grown to OD_600_ ∼0.6. Protein expression was induced with the addition of isopropyl-d-thiogalactopyranoside to a final concentration of 1 mM. Post induction cells were pelleted by centrifugation in a Beckman Avanti J-20 centrifuge (5050 × *g*, 12 min, 4°C) using a JLA-8.1000 rotor and resuspended in nickel affinity binding buffer (20 mM Tris-HCl pH 7.5, 500 mM NaCl, 5 mM imidazole) containing 1 mM PMSF. Cells were lysed by sonication on ice, centrifuged (12,500 × *g*, 30 min, 4°C) in a Sigma 3k30 centrifuge to remove debris and the cellular supernatant 0.45 μm syringe filtered. Lysate was loaded onto a 5-mL HisTrap FF column preequilibrated in binding buffer and eluted with a linear gradient of 5–250 mM imidazole. Fractions were analyzed by SDS polyacrylamide gel electrophoresis (SDS-PAGE) and pooled. Excess nickel was chelated with 5 mM ethylenediaminetetraacetic acid followed by dialysis into TBS buffer (25 mM Tris-HCl pH 7.5, 150 mM NaCl) at 4°C. Proteins were further purified using gel filtration chromatography on a HiLoad Superdex 200 26/60 column (for full-length PyoS2 constructs) or a HiLoad Superdex 75 26/60 (for PyoS2_NTD_ constructs), equilibrated in TBS buffer with eluted fractions analyzed and pooled accordingly.

### Oxidation, reduction, and alkylation of PyoS2 disulfide mutants

PyoS2 disulfide mutants were diluted to appropriate concentrations with TBS buffer, followed by treatment with 1,1′-Azobis(*N*,*N*-dimethylformamide; Diamide) or dl-dithiothreitol (DTT) to final concentrations of 1 and 5 mM, respectively, followed by incubation at room temperature for 45 min. To prevent disulfide reformation, reduced PyoS2 disulfide mutant proteins were desalted into denaturing buffer (25 mM Tris-HCl, pH 7.5, 150 mM NaCl, 6 M guanidine-HCl) and incubated at room temperature for 30 min. Proteins were then alkylated with 200 μM iodoacetamide for 1 h at room temperature, and subsequently desalted back into TBS buffer, prior to usage.

### AlexaFluor^488^ labeling of PyoS2 disulfide mutants

Ten micromolar PyoS2 disulfide mutants were treated with diamide or DTT, as described previously. Proteins were subsequently desalted into denaturing buffer (20 mM Tris-HCl pH 7.5, 150 mM NaCl, 6 M guanidine-HCl) and incubated for 30 min at room temperature. Proteins were then labeled by addition of 50 μM AlexaFluor^488^ C5-maleimide (5-fold molar excess) for 1 h at 37°C. Proteins were desalted into TBS buffer to remove excess fluorophore and analyzed by SDS–PAGE.

### Protein concentrations and AlexaFluor^488^ labeling efficiency

Protein concentration was determined by absorbance at 280 nm using an Eppendorf Biophotometer. Molecular weights and molar extinction coefficients for proteins were determined utilizing Expasy ProtParam Tool. Calculation of AlexaFluor^488^ labeling efficiency was estimated spectrophotometrically through measurement of absorption at 280 nm and *A*_494 nm_ (AlexaFluor^488^, *ɛ*_max_ = 71,000 cm^−1^ M^−1^, and pyoS2^NTD^  *ε*_280 nm_ = 16,390 cm^−1^ M^−1^) and concentrations determined using a correction factor for absorption at 280 nm by the fluorophore (AlexaFluor^488^, *A*_280 nm_ = 0.11 × *A*_488 nm_).

### Circular dichroism of PyocinS2_NTD_ constructs

PyoS2_NTD_ disulfide mutants were treated as described previously to generate oxidized and reduced (+alkylated) proteins. Proteins were dialyzed into 10 mM potassium phosphate buffer pH 7.5, and concentration was adjusted to 0.1 mg/mL. Far-UV spectra were obtained using a Jasco J-815 Spectropolarimeter over a wavelength range of 260–190 nm (with a digital integration time of 1 s and a 1-nm bandwidth). Protein-melting temperatures (*T*_m_) were determined by monitoring ellipticity at 222 nm as a function of temperature from 20 to 86°C.

### Pyocin plate cytotoxicity assays

*P. aeruginosa* strains were cultured overnight in LB at 37°C. Overnight cultures were used to inoculate (1:100) 10 mL fresh LB and incubate at 37°C to an OD_600_ ∼0.6. Bacterial lawns were prepared by adding 200 μL culture to 10 mL of molten soft LB-agar (0.7% [w/v] agar in LB) at 50°C and were poured onto LB-agar plates. Once set and dry, 2 μL of 3-fold serially diluted PyoS2 constructs ranging from 10 μM to ∼57 pM was applied to the lawn. Plates were incubated overnight at 37°C, and cytotoxicity was determined through observation of clearance zones.

### Pyocin kinetic time-course cytotoxicity assays

*P. aeruginosa* strain YHP17 was cultured as above in 10 mL LB to an OD_600_ ∼0.4 at 37°C. Ten nanomolar of PyoS2 construct was added, and culture was incubated at 37°C. After 0, 2, 5, 10, 15, 20, 25, 30, 45, and 60 min incubation with pyocin, 900 μL sample of culture was taken and added to 100 μL of trypsin to final concentration of 1 mg/mL in a stock buffer of 20 mM CaCl_2_, 0.1 M HCl pH 3.0. Samples were further incubated with the trypsin at 37°C for 30 min. One hundred microliters of each time point were diluted in LB between 10^0^- and 10^8^-fold, before spotting 3 μL of dilution range onto LB-agar plates, followed by incubation O/N at 37°C. CFU/mL values were calculated and plotted as a pseudo-first order model for translocation against time. The translocation rate constant was calculated from the fit gradient allowing calculation of the *t*_1/2_ (*t*_1/2_ = ln2/*k*). These experiments were performed in triplicate to generate standard errors.

### PyoS2 disulfide cytotoxicity assay

*P. aeruginosa* strain YHP17 was cultured as above in 10 mL LB to an OD_600_ ∼0.4 at 37°C. This was then split into 3 × 3 mL culture tube triplicates. Ten nanomolars of PyoS2 and PyoS2 disulfide mutants (oxidized and reduced [+alkylated], as described previously) were added to the triplicates, and tubes were incubated at 37°C. OD_600_ readings were taken at 30 min time points for 180 min, and resultant growth curve was plotted to evaluate cytotoxic activity. After 180 min, 1 mL of culture was taken and serially diluted between 10^0^- and 10^6^-fold with LB, and 50 μL plated out onto LB-agar plates. Plates were incubated at 37°C for 16 h, and the resulting colonies were counted and utilized to calculate YHP17 CFU/mL per culture.

### Fluorescence microscopy of *P. aeruginosa* with PyoS2_NTD_-AF488

Overnight cultures of *P. aeruginosa* were inoculated into M9-glucose media (6.78 g/L Na­_2_HPO_4_, 3 g/L KH_2_PO_4_, 0.5 g/L NaCl, 10 mM d-glucose, 1 mg/mL NH_4_Cl, 2 mM MgSO_4_) and incubated at 37°C. Once OD_600_ of 0.6 was reached, 1 mL culture was pelleted by centrifugation (7,000 × *g*, 3 min) followed by resuspension in 1 μM PyoS2_NTD_^-^AF488 in M9-glucose, with subsequent incubation at room temperature for 15 min in the dark. Cells were washed to remove excess label through pelleting and resuspension in M9-glucose three times. After final resuspension into an appropriate volume of M9-glucose (10–100 μL), 10 μL of cellular suspension was loaded onto 1% (w/v) agarose pads in M9-glucose on a microscopy slide followed by sealing with a clean glass cover slip. All images were collected on an Oxford Nanoimager S microscope at 100 ms exposure. For every image, 200 frames were collected and averaged. Green fluorescence was measured at 35% A474 laser power.

The data were analyzed through *z*-stack, and cell identification in trans-illumination images thresholded to generate a binary image of the cells. Regions of interest were subsequently transferred to images taken in the fluorescence channel and the average intensity in the regions of interest was obtained. A minimum of 50 bacterial cells were quantified for each sample. Student’s t tests were performed to determine *P*-values.

## Supplementary Material

pgae124_Supplementary_Data

## Data Availability

All data needed to evaluate the conclusions in the paper are present in the paper and the Supplementary material.
