# Supplementary material for: Structural constraints of pyocin S2 import through the ferripyoverdine receptor FpvAI
Source: PNAS Nexus. 2024 Mar 27;3(4):pgae124. doi: 10.1093/pnasnexus/pgae124 (PMC10994204; doi:10.1093/pnasnexus/pgae124)
Supplement: pgae124_Supplementary_Data [file pgae124_supplementary_data.zip › Goult_SI_Appendix_v2.pdf]

Supplemental Figure S1

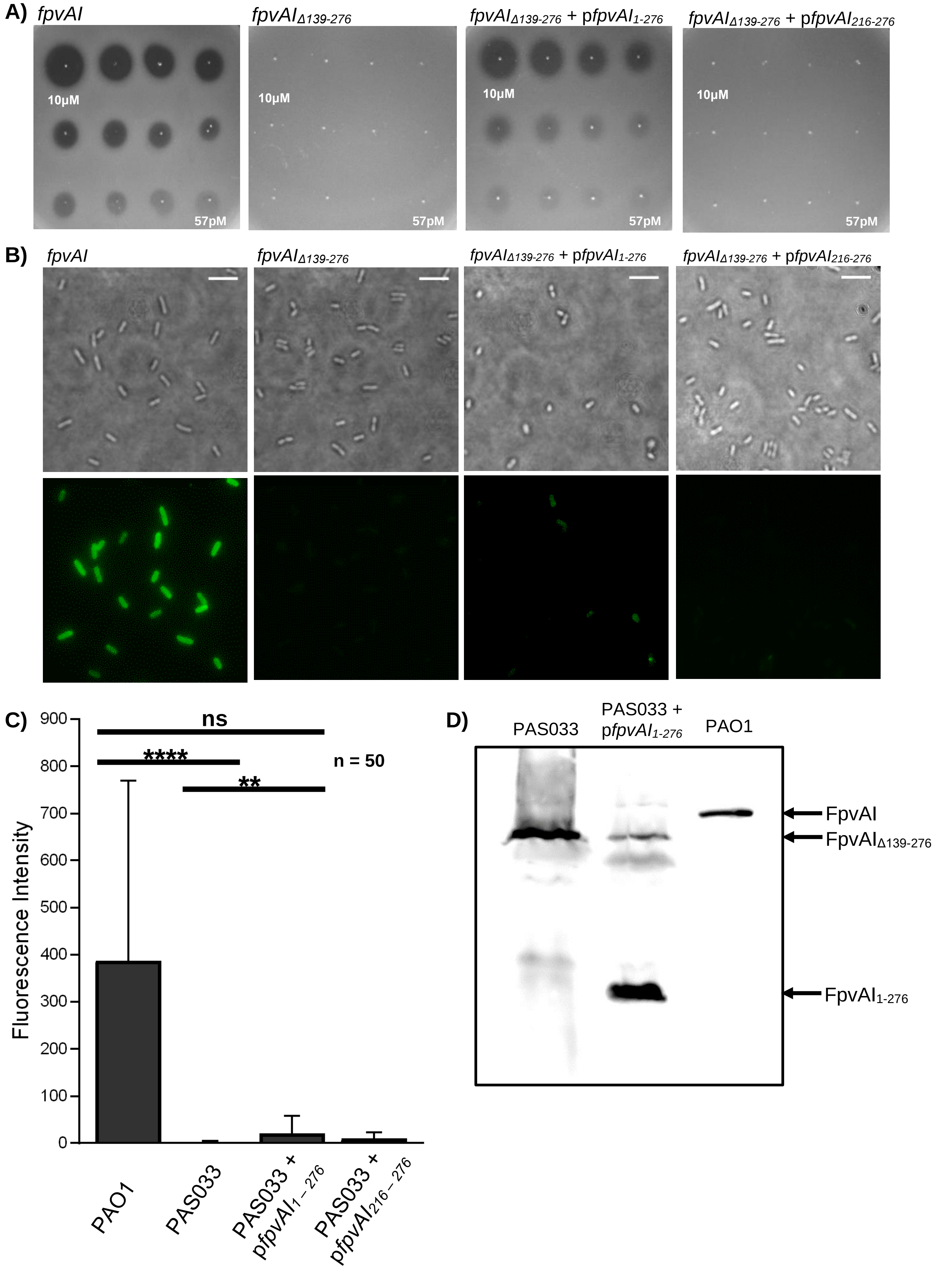

Figure S1: An intact FpvAI plug is required for PyoS2 cytotoxicity in vivo.

A) PyoS2 cytotoxicity assays show *P. aeruginosa* strain PAS033 ($fpvAI_{\Delta 139\text{-}276}$) is resistant to PyoS2E2 activity, compared to parent strain PAO1 (*fpvAI*). Expression of soluble FpvAI plug (p$fpvAI_{1\text{-}276}$) in PAS033 restores cytotoxic susceptibility, whereas expression of soluble non-labile plug subdomain alone (p$fpvAI_{216\text{-}276}$) does not restore cytotoxic susceptibility. Zones of clearance indicate cell killing. B) Fluorescent labelling of live *P. aeruginosa* strains (PAO1, PAS033 and PAS033 transformed with plasmids containing either p$fpvA_{1\text{-}276}$ or p$fpvA_{216\text{-}276}$) with $PyoS2_{NTD}$-AF488. The PAS033 strain exhibits no labelling compared to PAO1. Fluorescence labelling with $PyoS2_{NTD}$-AF488 is restored upon expression of the soluble FpvAI plug ($FpvAI_{1\text{-}276}$) but not through expression of the soluble non-labile plug subdomain alone. Scale bar, 5 μm. C) Quantification of fluorescence intensity of *P. aeruginosa* strains (n = 50 cells) labelled with $PyoS2_{NTD}$-AF488. **** and ** indicate *p*-values below 0.0001 and 0.01 in Student's *t*-test. The difference in $PyoS2_{NTD}$-AF488 labelling between PAO1 and PAS033 expressing p$fpvAI_{1\text{-}276}$ is not statistically significant. Similarly, the difference in $PyoS2_{NTD}$-AF488 labelling between PAS033 and PAS033 expressing p$fpvAI_{216\text{-}276}$ was found not to be statistically significant. Three independent biological repeats with a minimum of 50 bacterial cells each were quantified per sample. D) Western Blot of total membrane extracts of *P. aeruginosa* strains PAO1 (*fpvAI*), PAS033 ($fpvAI_{\Delta 139\text{-}276}$) and PAS033 + p$fpvAI_{1\text{-}276}$. Total cell membranes, prepared from cells grown for 16 h in succinate medium at 30 °C, were subjected to SDS-PAGE in a 12% polyacrylamide gel followed by transfer onto a PVDF membrane. FpvAI expression was detected with primary rabbit αFpvA antiserum. Expression of the non-labile plug subdomain alone could not be detected by this method.

Supplemental Figure S2

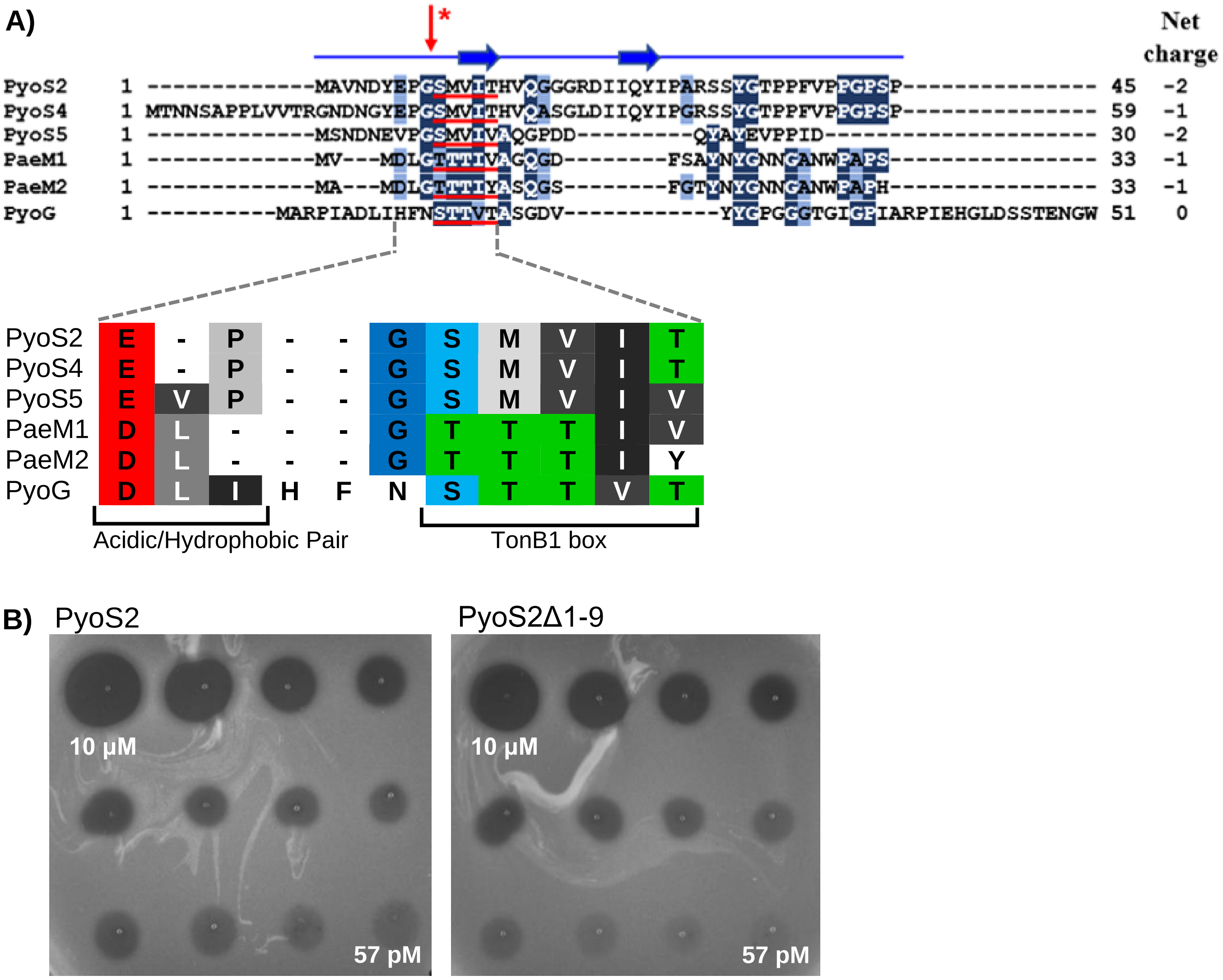

Supplemental Table S1

Protein	TonB1 Box Sequence	pI of residues preceding TonB1 box
PyoS2	SMVIT	3.67
PyoS4	SMVIT	4.37
PyoS5	SMVIV	3.67
PaeM1	TTTIV	3.80
PaeM2	TTTIY	3.80
PyoG	STTVT	6.50

Figure S2: Conservation of residues and charge across TonB box sequences in S-Type Pyocin family.

A) Sequence alignment of unstructured residues of known TonB1 dependent pyocins with the net charge of their predicted unstructured N-termini at pH 7.0. Red arrow denotes the PyoS2Δ1-9 truncation site. Alignment of unstructured N-terminal sequences of six S-type pyocins demonstrates the presence of an acid/hydrophobic pair preceding the TonB1 box in most cases. This is closely followed by the TonB1 box, a pentapeptide sequence containing a significant proportion of β-branched amino acids. Amino acids are colored based on their characteristics: acidic (*red*), hydrophobic (*black and grey*), serine (*cyan*), threonine (*green*) and glycine (*blue*). B) YHP17 plate killing assays demonstrate PyoS2Δ1-9 displays no significant reduction in cytotoxicity compared to PyoS2. Zones of clearance indicate cell killing.

Table S1: Theoretical isoelectric points of disordered regions preceding TonB1 box of S-type pyocins. pIs calculated using ExPasy (https://web.expasy.org/compute_pi)

Supplemental Figure S3

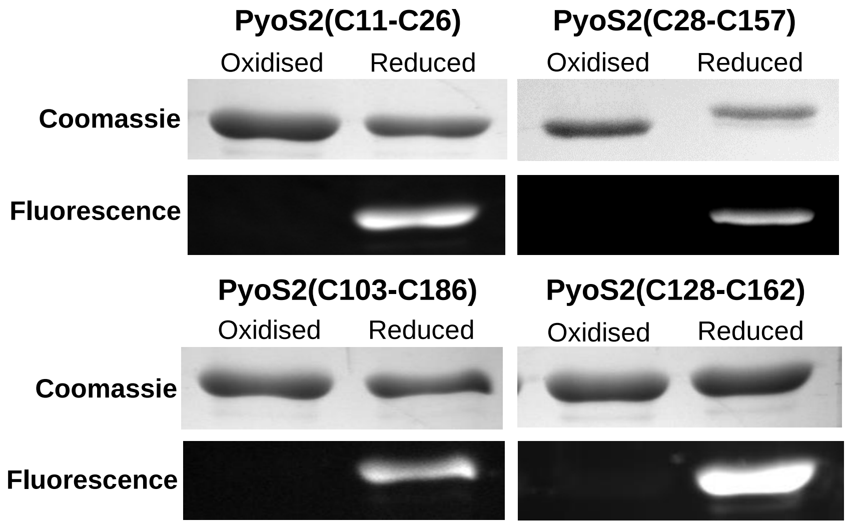

Figure S3: Evaluation of PyoS2 Disulfide Bond formation in vitro.

Confirmation of PyoS2 disulfide bond formation in vitro through AF488 fluorophore labelling of free thiols under different oxidation conditions. No labelling of free cysteines occurs in the oxidized samples, confirming disulfide bond formation.

Supplemental Figure S4

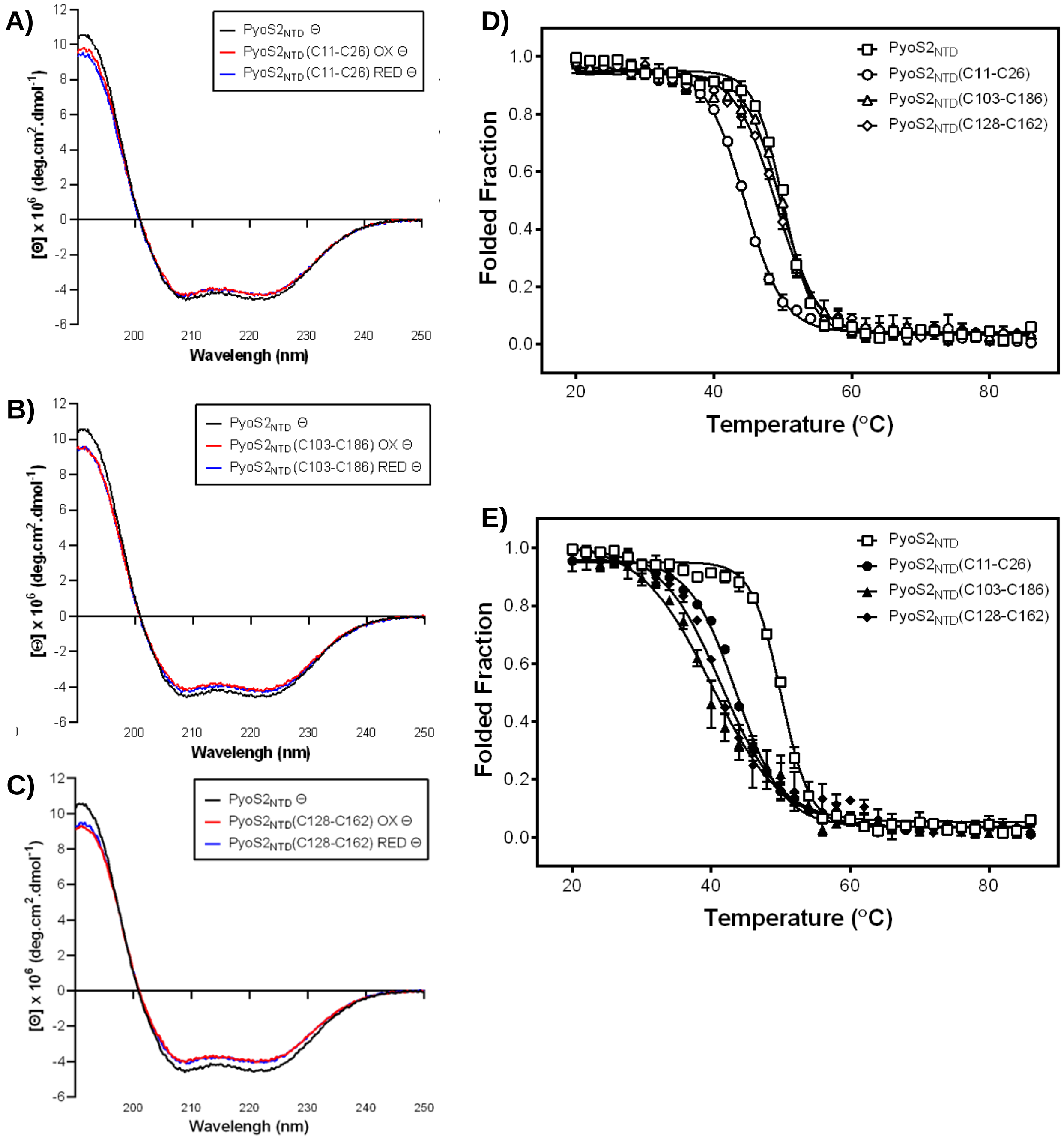

Figure S4: Disulfide bond formation within the $PyoS2_{NTD}$ does not increase the thermal stability (T_m) of the domain.

Far-UV CD spectrum of oxidized $PyoS2_{NTD}$ disulfide mutant A) $PyoS2_{NTD}$(C11-C26), B) $PyoS2_{NTD}$(C103-C186) and C) $PyoS2_{NTD}$(C128-C162) exhibit the double minima at 208 nm and 222 nm, characteristic of an α-helical secondary structure, akin to that of the N-terminal domain of wild-type PyoS2. There are minimal alterations in α-helical secondary structure upon reduction of the disulfide bond. D) Thermal melting curves for isolated N-terminal

domains of PyoS2 (□), PyoS2(C11-C26) (○), PyoS2(C103-C186) (Δ) and PyoS2(C128-C162) (◇) after disulfide oxidation obtained by far-UV CD spectroscopy E) Thermal melting curves for isolated N-terminal domains of PyoS2 (□), PyoS2(C11-C26) (●), PyoS2(C103-C186) (▲) and PyoS2(C128-C162) (◆) after disulfide reduction obtained by far-UV CD spectroscopy. The normalized average from 3 independent experiments was plotted against temperature with error bars representing the standard deviation.

Supplemental Figure S5

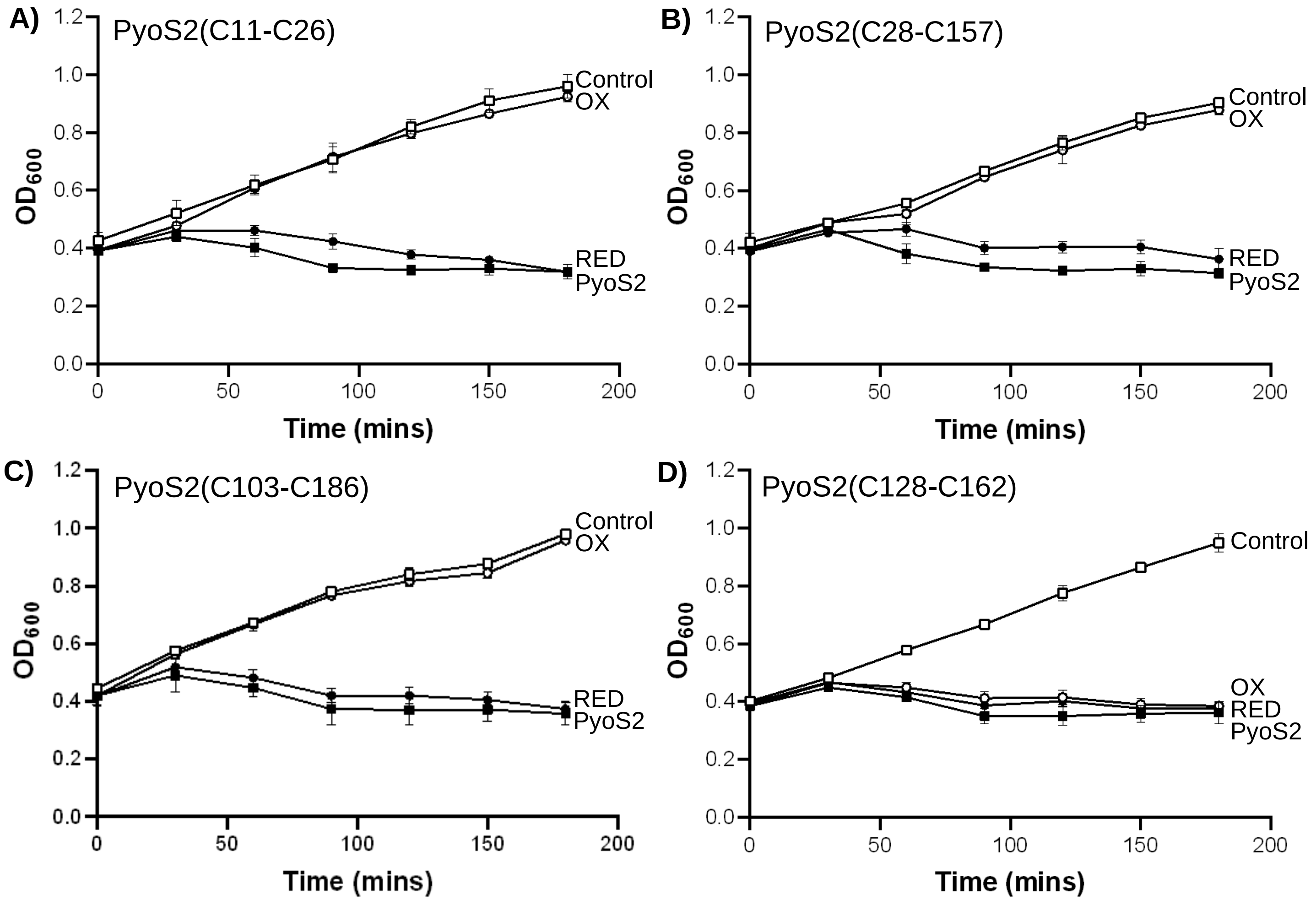

Figure S5: Introduction of disulfides into PyoS2 abolishes cytotoxic activity in a position-dependent manner.

YHP17 liquid culture killing assays for A) PyoS2(C11-C26) B) PyoS2(C28-C157) C) PyoS2(C103-C186) and D) PyoS2(C128-C162) demonstrate that disulfide bond formation inhibits PyoS2 cytotoxicity for disulfide positions C11-C26, C28-C157 and C103-C186. Disulfide position C128-C162 remains active in its oxidized form, suggesting it is still able to translocate through FpvAI. OD_{600} measurements averaged from six independent cultures with error bars representing the standard deviation.

Supplemental Movie S1: Simulation of PyoS2$_{NTD}$ import through the FpvAI transporter.

Steered molecular dynamic simulations (utilizing the FpvAI –PyoS2$_{NTD}$ crystal structure (PDB 5ODW)) of PyoS2$_{NTD}$ (*blue*) translocation through the FpvAI transporter pore. The FpvAI pore lumen, formed upon force-induced removal of the labile subdomain, is comprised of the β-barrel domain (*grey*) and the non-labile plug subdomain (*green*). The labile plug subdomain (residues 45 – 215) was excluded from these simulations.

Supplemental Movie S2: Simulation of PyoS2$_{NTD}$(C11-C26) import through the FpvAI transporter.

Steered molecular dynamic simulations of PyoS2$_{NTD}$(C11-C26) (*blue*) translocation through the FpvAI transporter pore. The FpvAI pore lumen, formed upon force-induced removal of the labile subdomain, is comprised of the β-barrel domain (*grey*) and the non-labile plug subdomain (*green*). Disulfide bond C11-C26 is highlighted as spheres (*yellow*) during translocation. The labile plug subdomain (residues 45 – 215) was excluded from this simulation.

Supplemental Movie S3: Simulation of PyoS2$_{NTD}$(C28-C157) import through the FpvAI transporter.

Steered molecular dynamic simulations of PyoS2$_{NTD}$(C28-C157) (*blue*) translocation through the FpvAI transporter pore. The FpvAI pore lumen, formed upon force-induced removal of the labile subdomain, is comprised of the β-barrel domain (*grey*) and the non-labile plug subdomain (*green*). Disulfide bond C28-C157 is highlighted as spheres (*yellow*) during translocation. The labile plug subdomain (residues 45 – 215) was excluded from this simulation.

Supplemental Movie S4: Simulation of PyoS2$_{NTD}$(C103-C186) import through the FpvAI transporter.

Steered molecular dynamic simulations of PyoS2$_{NTD}$(C103-C186) (*blue*) translocation through the FpvAI transporter pore. The FpvAI pore lumen, formed upon force-induced removal of the labile subdomain, is comprised of the β-barrel domain (*grey*) and the non-labile plug subdomain (*green*). Disulfide bond C103-C186 is highlighted as spheres (*yellow*) during

translocation. The labile plug subdomain (residues 45 – 215) was excluded from this simulation.

Supplemental Movie S5: Simulation of PyoS2$_{NTD}$(C128-C162) import through the FpvAI transporter.

Steered molecular dynamic simulations of PyoS2$_{NTD}$(C128-C162) (*blue*) translocation through the FpvAI transporter pore. The FpvAI pore lumen, formed upon force-induced removal of the labile subdomain, is comprised of the β-barrel domain (*grey*) and the non-labile plug subdomain (*green*). Disulfide bond C128-C162 is highlighted as spheres (*yellow*) during translocation. The labile plug subdomain (residues 45 – 215) was excluded from this simulation.
